# Supplementary material for: Chemerin and Chemokine-like Receptor 1 Expression in Ovarian Cancer Associates with Proteins Involved in Estrogen Signaling
Source: Diagnostics (Basel). 2023 Mar 2;13(5):944. doi: 10.3390/diagnostics13050944 (PMC10001027; doi:10.3390/diagnostics13050944)
Supplement: Supplementary file 1 [file diagnostics-13-00944-s001.zip › diagnostics-2230206-supplementary.pdf]

Supplementary figures

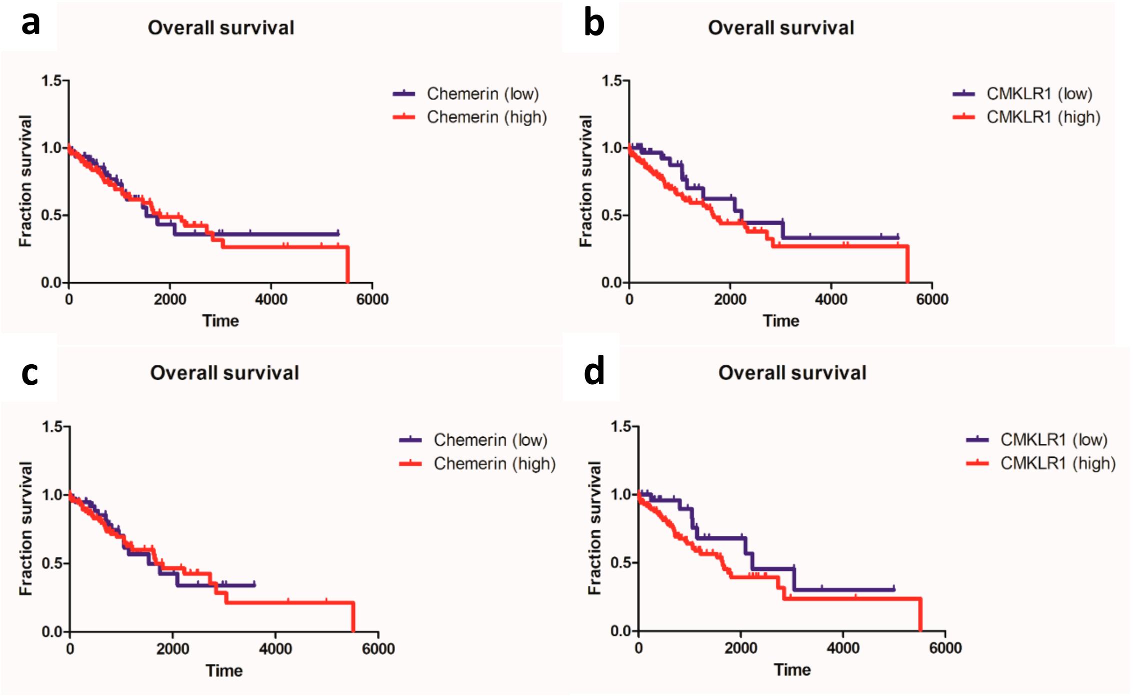

Supplementary Figure S1. Kaplan-Meier analyses of overall survival (OS) for chemerin (**a**) and CMKLR1 (**b**) low and high expression in all subtypes of ovarian cancer (OC) and chemerin (**c**) and CMKLR1 (**d**) low and high expression in serous OC.
